# Study on the effect of magnesium on leaf metabolites, growth and quality of tea tree

**DOI:** 10.3389/fpls.2023.1192151

**Published:** 2023-09-08

**Authors:** Ying Zhang, Qi Zhang, Yuhua Wang, Shaoxiong Lin, Meihui Chen, Pengyuan Cheng, Mengru Du, Xiaoli Jia, Jianghua Ye, Haibin Wang

**Affiliations:** ^1^ College of Tea and Food, Wuyi University, Wuyishan, China; ^2^ College of Horticulture, Fujian Agriculture and Forestry University, Fuzhou, China; ^3^ College of Life Science, Fujian Agriculture and Forestry University, Fuzhou, China; ^4^ College of Life Science, Longyan University, Longyan, China

**Keywords:** tea tree, magnesium, metabolomics, photosynthesis, quality

## Abstract

Magnesium (Mg) is one of the essential elements for the growth of tea trees. In this study, we investigated changes in metabolites, photosynthetic fluorescence parameters and quality indexes of tea leaves under different concentrations of magnesium treatment, and the results showed that there were no significant differences in the quantity and total content of metabolites in tea leaves under different Mg concentrations. The results of volcano map analysis showed that the content of 235 metabolites in tea leaves showed an increasing trend and the content of 243 metabolites showed a decreasing trend with the increase of Mg concentration. The results of the combined analysis of the OPLS-DA model and bubble map showed that 45 characteristic metabolites were screened at different concentrations of Mg. Among these, the content of 24 characteristic metabolites showed an increasing trend and 21 characteristic metabolites showed a decreasing trend with the increase of Mg concentrations. The results of KEEG pathway enrichment showed that 24 characteristic metabolites with a upward trend were significantly enriched in saccharides metabolism, nucleic acid metabolism and vitamin metabolism, while the 21 characteristic metabolites with a downward trend were enriched in the synthesis of plant secondary metabolites, phenylpropanoid biosynthesis, biosynthesis of terpenoids, synthesis and metabolism of alkaloids, and synthesis and metabolism of amino acids. It can be inferred that Mg regulation was beneficial to enhance the photosynthetic capacity of tea trees, improve the accumulation and metabolism of carbohydrate substances in tea trees, and thus promoted the growth of tea trees, but was not conducive to the synthesis of secondary metabolites and amino acids related to tea quality. The results of photosynthetic fluorescence parameters and quality indexes of the tea tree confirmed the conclusion predicted by metabolomics. This study provided a reference for regulating of the growth and quality of tea trees with Mg fertilizer in tea plantations.

## 1 Introduction

Magnesium (Mg), one of the 16 elements required for plant growth, is one of the most abundant macronutrients in plants and one of the most abundant cations in living cells (Javed et al., 2022). Mg acts as a cofactor for a range of enzymes involved in various physiological and biochemical processes such as chlorophyll synthesis, photosynthesis, and nucleic acid metabolism in plants, directly affecting the synthesis of plant proteins, photosynthesis and the development of cellular tissue structure (Kleczkowski and Igamberdiev, 2021). Mg was extremely important for plant growth, and Mg deficiency led to lower plant productivity and yield (Kwon et al., 2019). Mg regulation could effectively improve the absorption of macrotrophic elements by plants, which in turn promoted plant growth and increased plant yield (Kanjana, 2020). Therefore, Mg was extremely important in plant growth, and effective regulation of Mg supply during plant growth was of great significance.

China is the country with the largest tea plantation area and the highest production. Tea industry is an important agricultural industry in China and has an important contribution to the development of Chinese agriculture. Tea trees are mainly harvested from young buds and leaves, so a large amount of fertilizer is used in the cultivation process (Ye et al., 2023). Therefore, many scientists have carried out a large number of studies on the effects of fertilizer on the growth of tea tree, but mainly focused on the effects of N, P and K on tea growth and quality, and achieved important results, while fewer studies have been conducted on the effects of Mg on the growth of tea trees (Lin et al., 2022; Wei et al., 2022; Tang et al., 2023). Mg was reported to be extremely important for plant growth and had a greater effect on soil and nutrient concentrations than nitrogen or calcium fertilizers (Esteves et al., 2022). Increasing Mg supply in crops planted in Mg-deficient areas tended to improve crop quality, particularly when quality formation depended on Mg-driven photosynthesis and assimilated transport within plants, while excessive amounts of Mg did not improve yield and quality (Gerendás and Führs, 2013). For tea trees, Mg is extremely important for their growth, and the most significant change in the element content of tea leaves before and after pruning was Mg (Liu et al., 2022). Secondly, Mg could improve the photosynthetic capacity of tea trees, promote tea tree growth and increase tea yield (Li et al., 2021). It can be seen that Mg played an important role in the growth process of tea tree. However, metabolites were the end products of physiological changes in tea trees after Mg regulation, and few studies have been reported on how changes in metabolites of tea leaves are affected after changes in tea growth under Mg regulation. It is important to reveal the effect of Mg on metabolites of tea tree, for the rational use of Mg fertilizer in the process of tea tree planting, and then regulate the yield and quality of tea trees.

Accordingly, in this study, the tea tree was used as the research object, and the concentration of Mg in the culture solution was adjusted by hydroponics. At the same time, leaves of tea trees treated with different concentrations of Mg were collected, and their metabolites were determined by ultra performance liquid chromatography tandem mass spectrometry (UPLC-ESI-MS/MS), and the metabolic mechanism of tea leaves in response to Mg regulation was analyzed to provide some guidance for the rational use of Mg fertilizer in tea plantations.

## 2 Materials and methods

### 2.1 Plant material

In this study, experimental research on Mg regulation was conducted using Wuyi Rougui tea seedlings as material. Specifically, one-year-old tea seedlings (35 cm in height and 0.3 cm in diameter) with uniform growth were selected, washed with water to remove root soil, and pre-cultured in a nutrient solution at pH 4.5 and electrical conductivity (EC) value of 0.55 ms/cm for 45 days to allow tea seedlings to recover and grow normally. During this period, oxygen pumps were used to ventilate the culture solution for 24 hours. The formulation of tea tree hydroponic nutrient solution was configured according to the method of Sun et al. (2020), and the nutrient solution mainly contained 125 μmol/L KNO_3_, 187.5 μmol/L (NH_4_)_2_SO_4_, 100 μmol/L KH_2_PO_4_, 25 μmol/L K_2_SO_4_, 100 μmol/L CaCl_2_, 100 μmol/L MgSO_4_, 16 μmol/L FeSO_4_, and 200 μmol/L AlSO_4_. Then the tea seedlings were taken out and the roots were rinsed with deionized water for 3 times, and the tea seedlings were transferred to the nutrient solution with different Mg concentrations for cultivation, with 3 replicates for each treatment. The pots for cultivating tea seedlings were 28 cm in length, 25 cm in width, 14 cm in height, 8 L of nutrient solution, 20 tea seedlings per pot. The nutrient solution with different Mg concentrations was configured according to the above method, while Mg concentrations in the nutrient solution were adjusted to 0 mmol/L, 0.4 mmol/L and 0.8 mmol/L, and concentrations of other ions were consistent with the above formulation. Tea seedlings were transplanted into nutrient solutions with different Mg concentrations for 21 days, and the nutrient solution was changed every 7 days. Tea seedlings were cultured in a greenhouse with 24 h of being continuously ventilating and 12 h of light (8:00 ~ 20: 00) per day, with a light intensity was 1500 lux, a temperature of 25 °C during the light period and 20 °C for the rest, and a humidity maintained at 75% ± 5%. After 21 days of treatment with different Mg concentrations, one bud and three leaves of tea seedlings were collected and immediately frozen in liquid nitrogen for metabolites and tea quality index determination in tea leaves.

### 2.2 Determination of photosynthetic fluorescence parameters in tea leaves

The chlorophyll content of tea leaves was determined by chlorophyll analyzer (SPAD-502 PLUS, Japan) with 5 replicates per treatment. The photosynthetic fluorescence parameters of tea leaves were determined by PAM-2500 chlorophyll fluorescence analyzer (WALZ, Germany) with 5 replicates per treatment, and the indexes were maximal fluorescence, maximal fluorescence under light, instantaneous chlorophyll fluorescence, maximum quantum efficiency of PSII, non-photochemical quenching coefficient, and actual photochemical efficiency of PSII. In the process of determination, tea leaves were acclimated to the dark environment for 30 min using a measurement light of less than 0.05 μmol·m^-2^·s^-1^ and a saturated pulse light of 8000 μmol·m^-2^·s^-1^.

### 2.3 Determination of tea quality index

The quality indexes of tea leaves were mainly determined by water extract, tea polyphenols, theanine, caffeine, flavone, free amino acid and soluble sugar, with 3 replicates per treatment. Tea polyphenols content was determined by folinol colorimetry according to the National Standard of the People’s Republic of China (GB/T 8313-2018) (General Administration of Quality Supervision, Inspection and Quarantine of the People's Republic of China, 2018). Theanine content was determined by high performance liquid chromatography (GB/T 23193-2017) (General Administration of Quality Supervision, Inspection and Quarantine of the People's Republic of China, 2017). Caffeine content was determined by ultraviolet spectrophotometry (GB/T 8312-2013) (General Administration of Quality Supervision, Inspection and Quarantine of the People's Republic of China, 2013a). The free amino acid content was determined by UV spectrophotometric method, with specific reference to the National Standard of the People’s Republic of China, GB/T 8314-2013 (General Administration of Quality Supervision, Inspection and Quarantine of the People's Republic of China, 2013b). The contents of water extract, flavonoids and soluble sugars were determined by hot water extraction method, aluminum trichloride colorimetric method and anthrone colorimetric method, respectively, according to Wang et al. (2020).

### 2.4 Determination of metabolites in tea leaves

#### 2.4.1 Sample preparation and extraction

A vacuum freeze dryer (Scientz-100F) was used to freeze dry the above tea samples. Freeze-dried samples were crushed with zirconia beads at 30 Hz for 1.5 min using a mixer mill (MM 400, Retsch) to form a lyophilized powder. 50 mg lyophilized powder was dissolved with 1.2 mL 70% methanol solution, and vortexed for 30s every 30 min for 6 times, and then the extracts were obtained after centrifugation at 12000 rpm for 3 min. The extracts were filtrated through disposable needle filters (SCAA-104, 0.22 μm pore size; ANPEL, Shanghai, China), and then analyzed by UPLC-MS/MS.

#### 2.4.2 UPLC conditions

The above sample extracts were analyzed by an UPLC-ESI-MS/MS system (UPLC, SHIMADZU Nexera X2; MS, Applied Biosystems 4500 QTRAP),. The column of UPLC was an Agilent SB-C18 (1.8 µm, 2.1 mm × 100 mm) and the mobile phase consisted of solvent A (pure water containing 0.1% formic acid) and solvent B (acetonitrile containing 0.1% formic acid). Sample measurements were performed with a gradient program using the starting conditions of 95% A and 5% B. Within 9min, a linear gradient to 5% A, 95% B was programmed, and held for 1 min. Subsequently, it was adjusted to a mixture of 95% A, 5.0% B within 1.1 min and held for 2.9 min. The injection volume of UPLC was 4 μL, the flow velocity was set to 0.35 mL/min, the column temperature was set to 40°C. The effluent was alternatively connected to an ESI-triple quadrupole-linear ion trap (QTRAP)-MS for subsequent determination.

#### 2.4.3 ESI-Q TRAP-MS/MS

Analysis conditions for electrospray ionization (ESI) sources were specified below. The source temperature was 550°C, the ion spray voltage (IS) was 5500 V (Positive ion mode)/-4500 V (Negative ion mode), ion source gas I (GSI), gas II(GSII), curtain gas (CUR) were set to 50, 60, and 25 psi, respectively, and the collision-activated dissociation(CAD) was higher. Instrument commissioning and mass calibration were performed with 10 and 100 μmol/L polypropylene glycol solutions in QQQ and LIT modes, respectively. QQQ scans were used as MRM experiments, and collision gas (Nitrogen) was set as the medium. Declustering potential (DP) and collision energy (CE) of each MRM transition were further optimized. Specific MRM transitions were monitored for each period based on metabolites eluted during this period.

### 2.5 Statistical analysis

Excel 2017 software was used to perform preliminary data processing and calculate the mean. IBM SPSS Statistics 21.0 software was used for T-text, F-text and correlation analysis. R version 4.2.3 software (main R libraries were gghalves, ggbiplot, ggplot2, ropls, clusterProfiler) was used to make cloud and rain maps, principal component maps, volcanic maps, orthogonal partial least squares discriminant analysis (OPLS-DA, package used for this was ropls and mixOmics), bubble map and KEEG metabolic pathway, Heml 1.0 software was used to make heat maps.

## 3 Results and discussion

### 3.1 Metabolomic analysis of tea leaves under Mg regulation

**Figure 1:**
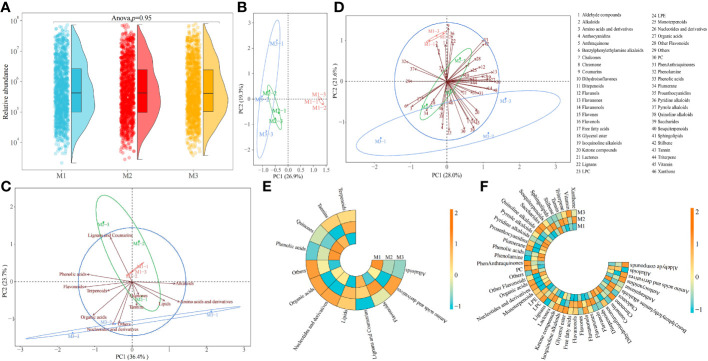
Metabolomic analysis of tea leaves under different Mg concentrations M1: Control; M2: The Mg concentration is 0.4 mmol/L; M3: The Mg concentration is 0.8 mmol/L; **(A)** Analysis of total metabolites content in tea leaves; **(B)** PCA analysis of metabolites in tea leaves; **(C)** PCA analysis of primary classification of metabolites in tea leaves; **(D)** PCA analysis of secondary classification of metabolites in tea leaves; **(E)** Heat map analysis of metabolites content in tea leaves in primary classification; **(F)** Heat map analysis of metabolites content in tea leaves in secondary classification.

Mg is one of the elements required for plant growth and plays an important regulatory role in the plant growth. Effective regulation of plant growth processes by Mg availability could alter the physiological mechanism of plants, affecting their physiological metabolism, which in turn led to changes in their metabolites (Ahmad et al., 2022; Hamedeh et al., 2022). In this study, ultra performance liquid chromatography tandem mass spectrometry (UPLC-ESI-MS/MS) was used to determine the changes in metabolites in tea leaves under different concentrations of Mg treatment, and the results showed (**Figure 1A**) that a total of 1240 metabolites were identified in tea leaves under different concentrations of Mg treatment, and secondly, there was no significant difference between the total metabolite contents of tea leaves under different Mg concentrations. The results of the principal component analysis of tea metabolites showed (**Figure 1B**) that the two principal components could distinguish three samples, in which the contribution rate of principal component 1 and principal component 2 was 26.9% and 19.3%, respectively, and the overall contribution rate was 46.2%. The 1240 tea metabolites were further classified and analyzed, of which the primary classification could be divided into 12 categories and the secondary classification could be divided into 46 categories. The results of the principal component analysis of the primary classification showed (**Figure 1C**) that the two principal components could distinguish three samples, in which the contribution rate of principal component 1 and principal component 2 was 36.4% and 23.7%, respectively, and the overall contribution rate was 61.1%. Secondly, the analysis showed that the control (M1) was located at the positive end of principal component 2, the Mg concentration of 0.8 mol/L (M3) was located at the negative end of principal component 2, and the Mg concentration of 0.4 mol/L (M2) was in the middle. The results of the principal component analysis of the secondary classification showed (**Figure 1D**) that the two principal components could distinguish three samples, in which the contribution rate of principal component 1 and principal component 2 was 28.0% and 21.6%, respectively, and the overall contribution rate was 49.6%. Secondly, the analysis found that similar to the PCA results of the primary classification, in the secondary classification, M1 was still located at the positive end of principal component 2, M3 was located at the negative end of principal component 2, and M2 was in the middle. It can be seen that after Mg treatment with different concentrations, although there was no significant difference in the total quantity and content of metabolites in tea leaves, there may be significant differences among different metabolites.

On this basis, this study further analyzed the changes in the classification of primary and secondary metabolites in tea leaves after Mg treatment with different concentrations. The results of the primary classification showed (**Figure 1E**) that with the increase of Mg concentration, the contents of alkaloids, lignans and coumarins, and phenolic acids showed a decreasing trend, and the contents of flavonoids, lipids, and terpenoids increased first and then decreased, and the contents of amino acids and derivatives, nucleotides and derivatives, organic acids, quinones and tannins decreased first and then increased, while others showed an upward trend. The results of secondary classification showed (**Figure 1F**) that with the increase of Mg concentration, the contents of alkaloids, coumarins, ditepenoids, phenolamine, phenolic acids and stilbene showed a decreasing trend, and the contents of benzylphenylethylamine alkaloids, chalcones, flavanols, flavanones, flavols, flavonols, free fatty acids, lactones, lignans, LPE, sesquiterpenoids, sphingolipids, tannin, triterpene and xanthone increased first and then decreased, and the contents of amino acids and derivatives, anthraquinone, dihydroisoflavones, glycerol ester, isoquinoline alkaloids, monoterpenoids, nucleotides and derivatives, organic acids, other flavonoids, plumerane, proanthocyanidins, pyridine alkaloids, pyrrole alkaloids and quinoline alkaloids decreased first and then increased, while the contents of aldehyde compounds, anthocyanidins, chromone, flavanonols, ketone compounds, LPC, PC, phenanthraquinones, saccharides and vitamin showed an increasing trend. It can be seen that there might be significant differences in the content of different metabolites in tea leaves after Mg treatment with different concentrations.

### 3.2 Comparative analysis of metabolites in tea leaves under magnesium regulation

**Figure 2:**
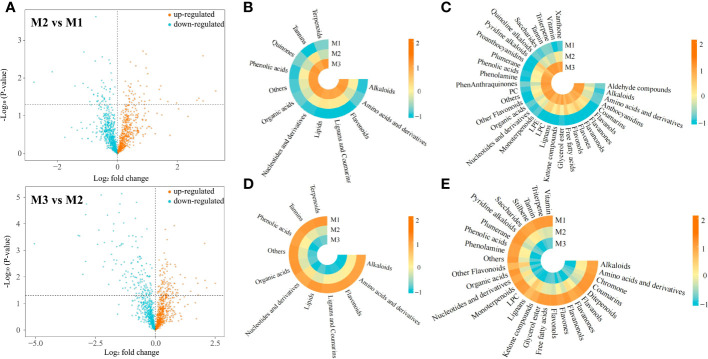
Comparative analysis of metabolites in tea leaves under different Mg concentrations M1: Control; M2: The Mg concentration is 0.4 mmol/L; M3: The Mg concentration is 0.8 mmol/L; **(A)** Volcanic map analysis of metabolites in tea leaves under different Mg concentrations; **(B)** Heat map of primary classification of metabolites with increased content under different Mg concentrations; **(C)** Heat map of secondary classification of metabolites with increased content under with different Mg concentrations; **(D)** Heat map of primary classification of metabolites with decreased content under different Mg concentrations; **(E)** Heat map of secondary classification of metabolites with decreased content under different Mg concentrations.

In this study, we analyzed the changes in metabolite content in tea leaves under different concentrations of Mg treatment, and the results showed (**Figure 2A**) that after 0.4 mmol/L Mg treatment (M2), the contents of 654 metabolites were up-regulated and 586 metabolites were down-regulated compared to the control (M1); after 0.8 mmol/L Mg treatment (M3), the contents of 662 metabolites were up-regulated and 578 metabolites were down-regulated compared to M2. Further analysis revealed that a total of 235 metabolites showed an upward trend and 243 metabolites showed a downward trend with the increase of Mg concentration (M1-M3). The 235 metabolites with an increasing trend could be divided into 12 categories by the primary classification, while 243 metabolites with a decreasing trend could be divided into 11 categories by the primary classification, and the category with the main difference was quinones (**Figures 2B, D**). The results of the secondary classification (**Figures 2C, E**) showed that 235 metabolites with an upward trend could be classified into 34 categories, while 243 metabolites with a downward trend could be classified into 29 categories, and the main differences were anthocyanidins, LPE, PC, phenanthraquinones, quinoline, alkaloids, proanthocyanidins, ditepenoids, stilbene. It can be seen that the contents of different metabolites in tea leaves changed significantly after Mg treatment with different concentrations.

### 3.3 Screening of key metabolites in tea leaves under magnesium regulation

**Figure 3:**
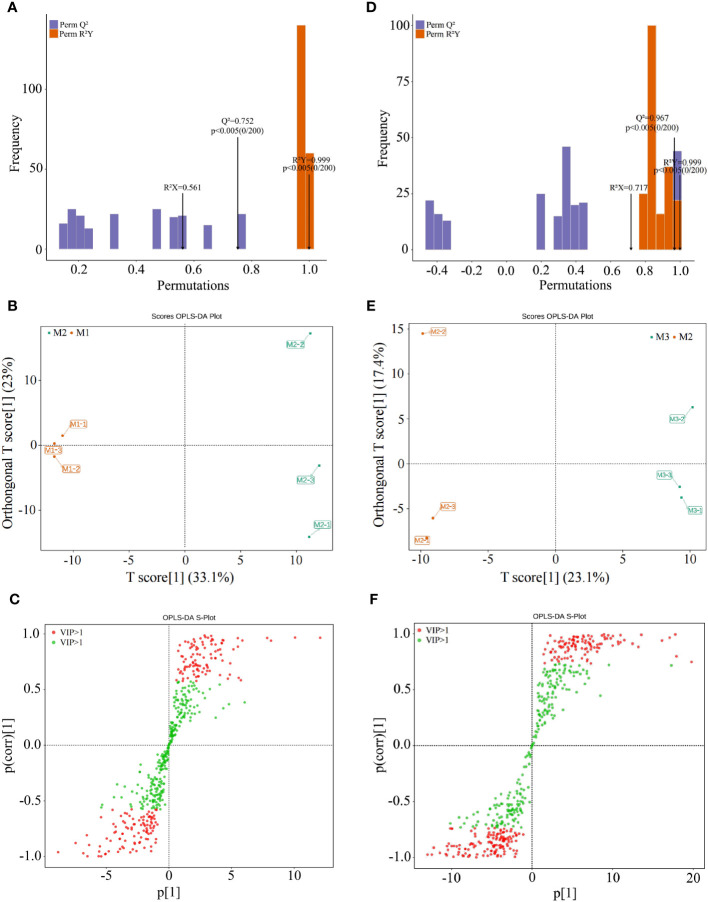
OPLS-DA model screening of key metabolites in tea leaves treated with different Mg concentrations M1: Control; M2: The Mg concentration is 0.4 mmol/L; M3: The Mg concentration is 0.8 mmol/L; **(A)** The OPLS-DA model for the fitting degree test of M2 and M1; **(B)** The OPLS-DA model for analysis of metabolites of M2 and M1; **(C)** The OPLS-DA loading diagram for metabolites of M2 and M1; **(D)** The OPLS-DA model for the fitting degree test of M3 and M2; **(E)** The OPLS-DA model for analysis of metabolites of M3 and M2; **(F)** The OPLS-DA loading diagram for metabolites of M3 and M2.

OPLS-DA can be used to model the correlation between metabolite content and sample class, and to screen markers that characterize sample differences by the variable importance projection value (VIP value) (Li et al., 2023). Meanwhile, to check the reliability of the OPLS-DA model, a permutation test is usually used to validate the model and thus assess its accuracy (Rivera-Pérez et al., 2022). Accordingly, on the basis of the above analysis, this study found that a total of 478 metabolites showed significant changes in content after treatment with different Mg concentrations, of which 235 metabolites showed an increasing trend and 243 metabolites showed a decreasing trend with the increase of Mg concentration (M1~M3). In order to screen and obtain the key compounds with major changes after Mg treatment with different concentrations, the OPLS-DA model was used for analysis in this study, and the results showed (**Figures 3A, D**) that the OPLS-DA model for M1 and M2 all had a R^2^Y value of 0.999 (*p* < 0.005) for goodness of fit and a predictive Q^2^ value of 0.752 (*p* < 0.005), while the OPLS-DA model for M2 and M3 had a goodness-of-fit R^2^Y value of 0.999 (*p* < 0.005) and a predictive Q^2^ value of 0.967 (*p* < 0.005). It can be seen that the R^2^Y and Q^2^ values of the two models reached significant levels, and the model fit was good and credible for further analysis.

**Figure 4:**
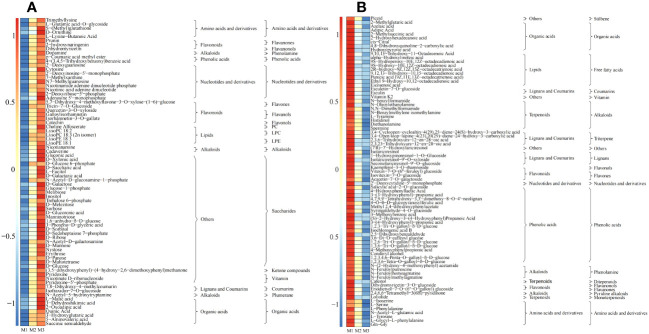
Content analysis of key metabolites screened by the OPLS-DA model M1: Control; M2: The Mg concentration is 0.4 mmol/L; M3: The Mg concentration is 0.8 mmol/L; **(A)** Heat map analysis of key metabolites with increased content under different Mg concentrations; **(B)** Heat map analysis of key metabolites with decreased content under different Mg concentrations.

The results of the OPLS-DA scoring chart showed (**Figures 3B, E**) that the OPLS-DA model could effectively distinguish M1, M2 and M3 two by two in different regions. There were significant differences in metabolites between M1, M2 and M3. The results of S-plot analysis showed (**Figures 3C, F**) that 202 key metabolisms (VIP >1) could distinguish M1 and M2, and 246 key metabolisms (VIP >1) could distinguish M2 and M3. Further analysis revealed that 154 key metabolites (VIP >1) could distinguish M1, M2 and M3; 75 metabolites showed an increasing trend in content with an increase of Mg concentration (M1-M3), and the top 4 categories with the highest amount were saccharides (37.33%), nucleotides and derivatives (12.00%), organic acids (9.33%), and amino acids and derivatives (6.67%) (**Figure 4A**); whereas 79 of 154 key metabolites showed a decreasing trend in content, and the top 4 categories with the highest number were phenolic acids (26.58%), free fatty acids (11.39%), alkaloids (10.13%), and organic acids (10.13%) (**Figure 4B**). Mg is an important component of plant chlorophyll and pigment, which can promote plant photosynthesis and improve the synthesis of carbohydrate and amino acids (Huang et al., 2021; Uçgun et al., 2022). At the same time, Mg is beneficial to promote plant metabolism, improve plant cell division ability and promote plant growth (Pacheco et al., 2020). Under Mg stress, plants had the ability to reduce primary metabolic capacity and enhance secondary metabolic capacity to resist the external environment, which in turn promoted the secretion of secondary metabolites and increased the content of secondary metabolites, especially phenols and alkaloids (Ahmad et al., 2022). Secondly, Mg deficiency also induced the increase in the content of fatty acids and some organic acids in plants (Anand et al., 2019). It can be seen that under Mg regulation, with the increase of Mg concentration, the photosynthesis capacity of tea tree was enhanced, the primary metabolic capacity of tea trees was improved, and the content of saccharides and organic acids in tea leaves were increased, and the metabolic capacity of nucleic acid of tea tree was increased, which in turn promoted the growth of tea trees. Secondly, when the Mg ion supply was sufficient, the tea tree would reduce secondary metabolic capacity and decrease the synthesis of secondary metabolites, especially phenolic acids and alkaloids, in the normal growth environment.

### 3.4 Screening of characteristic metabolites from key metabolites in tea leaves under magnesium regulation

**Figure 5:**
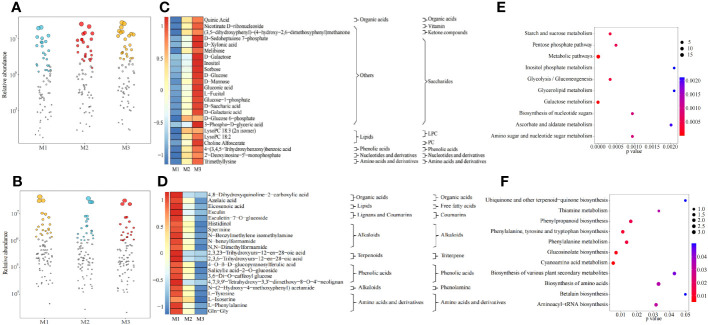
Characteristic metabolites screening from key metabolites M1: Control; M2: The Mg concentration is 0.4 mmol/L; M3: The Mg concentration is 0.8 mmol/L; **(A)** Bubble characteristics of key metabolites with increased content under different Mg concentrations; **(B)** Bubble characteristics of key metabolites with decreased content under different Mg concentrations; **(C)** Heat map analysis of characteristic metabolites with increased content under different Mg concentrations; **(D)** Heat map analysis of characteristic metabolites with decreased content under different Mg concentrations; **(E)** KEEG pathway analysis of 24 characteristic metabolites with increased content; **(F)** KEEG pathway analysis of 21 characteristic metabolites with decreased content.

Based on the above analysis, this study further analyzed the contents of 154 key metabolites that differentiated different Mg treatments. Bubble feature map analysis was performed with 154 key metabolites. The results showed (**Figure 5**) that the content of 45 characteristic metabolites accounted for more than 90% of the content of 154 key metabolites, of which 24 characteristic metabolites showed an increasing trend with the increase of Mg concentration (**Figure 5A**), while 21 showed a decreasing trend (**Figure 5B**). Secondly, the analysis found that the 24 characteristic metabolites with an increasing trend were mainly organic acids, vitamin, ketone compounds, saccharides, lipids, nucleotides and derivatives, phenolic acids, amino acids and derivatives, of which saccharides content accounted for more than 50% (**Figure 5C**); whereas the 21 characteristic metabolites with a decreasing trend were mainly organic acids, lipids, coumarins, alkaloids, triterpene, phenolic acids, amino acids and derivatives, of which alkaloids content reached more than 42% (**Figure 5D**). Further analysis revealed that the key changes in metabolites in tea leaves regulated by Mg were an increase in the content of vitamin, ketone compounds, saccharides, nucleotides and derivatives, while the content of coumarins, alkaloids and triterpene decreased.

Saccharides have been reported to be the main product of photosynthesis in plants, and an increase in saccharides content was beneficial to the enhancement of plant metabolism, which in turn increased plant growth rate, while nucleotides and derivatives could promote plant cell division and reproduction (Pacheco et al., 2020; Uçgun et al., 2022). Vitamins help protect plants from the harmful side effects of photosynthesis (Šola et al., 2021). In this study, KEEG pathway enrichment was performed on 24 characteristic metabolites whose content increased in tea leaves with increased Mg concentrations. The results showed (**Figure 5E**) that 24 characteristic metabolites were enriched into 29 metabolic pathways, of which 10 metabolic pathways were enriched to a significant level (*p* < 0.05). Further analysis showed that of the 10 metabolic pathways that were significantly enriched, 5 were related to saccharides metabolism, 1 was related to nucleic acid metabolism, and 1 was related to vitamin metabolism. It can be seen that Mg regulation was conducive to enhancing the photosynthetic capacity of tea trees, improving the accumulation and metabolism of carbohydrate substances in tea trees, and thus promoting the growth of tea trees.

Both yield and quality are very important for tea. Photosynthesis is beneficial to increase tea yield, but tea quality formation is closely related to the content of secondary metabolites and amino acid in tea leaves. Coumarins, alkaloids and triterpene were all secondary metabolites of plants. Secondly, coumarins, alkaloids and amino acids were important in tea taste formation, while triterpene was important for tea aroma formation (Zhao et al., 2020; Zhou et al., 2022). Livigni et al. (2019) also found that Mg promoted plant growth, but excessive Mg was detrimental to the synthesis of alkaloids, terpenoids and secondary phenylene metabolites. Liu et al. (2022) also found that secondary metabolites in tea trees increased when Mg content was low in the external environment. In this study, KEEG pathway enrichment was further performed on 21 characteristic metabolites whose content decreased in tea leaves with increased Mg concentrations, and the results showed (**Figure 5F**) that 21 characteristic metabolites were enriched into 28 metabolic pathways, and 11 metabolic pathways reached a significant level (*p* < 0.05). Further analysis showed that the 10metabolic pathways that were significantly enriched were mainly the synthesis of plant secondary metabolites, phenylpropanoid biosynthesis, biosynthesis of terpenoids, alkaloid synthesis and metabolism, and amino acid synthesis and metabolism. It can be seen that magnesium regulation was not conducive to the synthesis of secondary metabolites and amino acids in tea leaves, and was not conducive to the improvement of tea quality.

### 3.5 Growth and quality index analysis of tea tree

**Figure 6:**
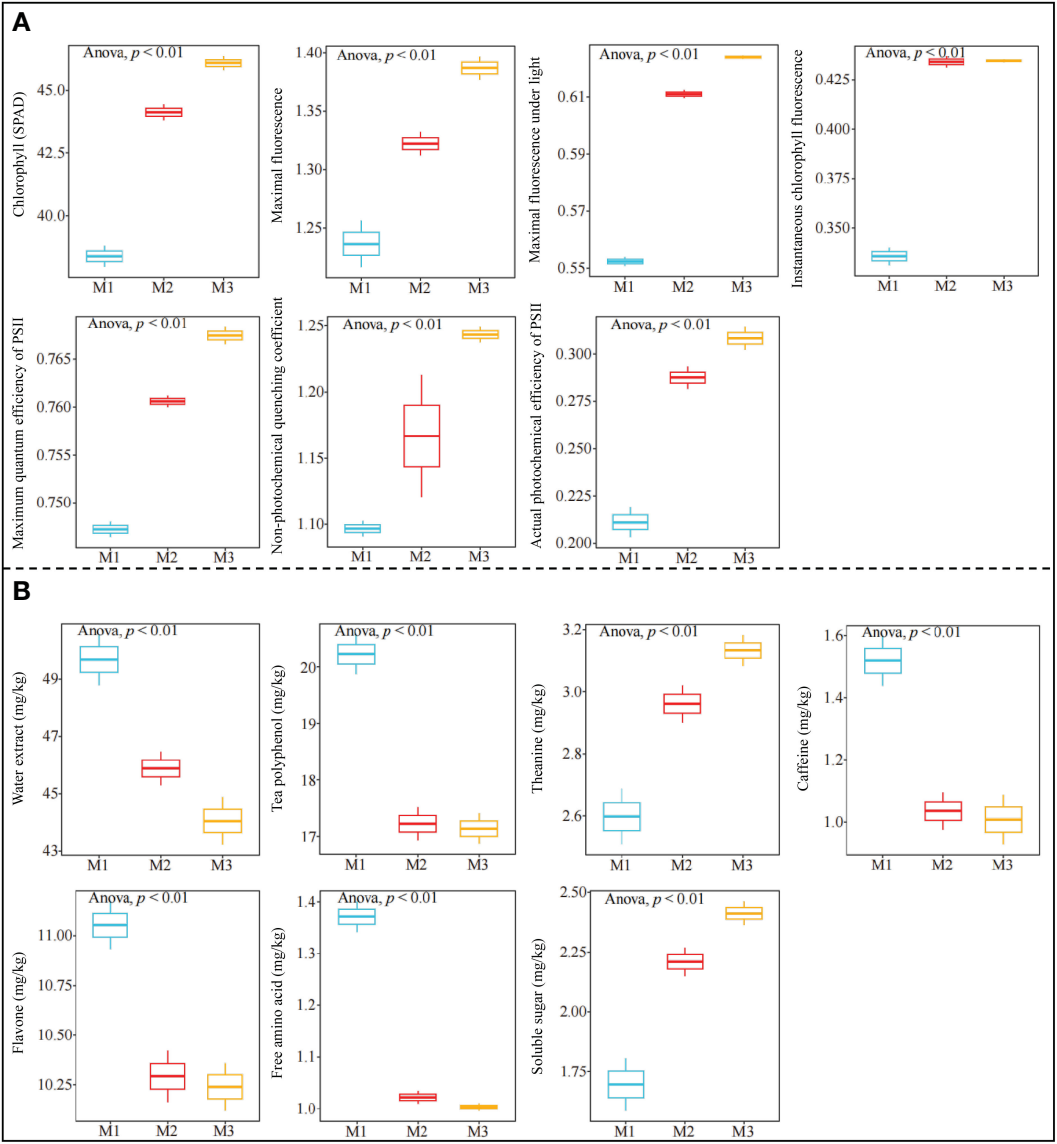
Effects of different Mg concentrations on growth and quality indexes of tea tree M1: Control; M2: The Mg concentration is 0.4 mmol/L; M3: The Mg concentration is 0.8 mmol/L; **(A)** Analysis of photosynthetic fluorescence parameters of tea tree under Mg regulation; **(B)** Analysis of tea quality index under Mg regulation.

The above metabolome study hypothesized that Mg regulation was beneficial to enhance photosynthetic capacity and improve accumulation and metabolism of carbohydrate substances in tea trees, which in turn promoted tea tree growth, but was detrimental to the synthesis of secondary metabolites related to tea quality. Previously, some scholars have also reported that magnesium regulation can improve the photosynthesis capacity of tea trees, thus increasing the biomass of tea trees, but it will have a certain impact on the synthesis of tea tree quality indexes (He et al., 2023; Zhang et al., 2023; Zhang et al., 2023). Accordingly, this study further determined the photosynthetic fluorescence parameters of tea leaves under different magnesium concentrations treatment, and the results showed (**Figure 6A**) that with the increase of treatment magnesium concentration, chlorophyll content, maximal fluorescence, maximal fluorescence under light, instantaneous chlorophyll fluorescence, maximum quantum efficiency of PSII, non-photochemical quenching coefficient and actual photochemical efficiency of PSII in tea leaves showed a significant upward trend. The results of the quality index analysis showed (**Figure 6B**) that the content of water extract, tea polyphenol, caffeine, flavone and free amino acid in tea leaves decreased significantly with the increase of Mg concentration, while the content of theanine and soluble sugar showed a significant upward trend. It can be seen that Mg regulation was beneficial to improve the photosynthetic capacity of tea trees, and then improved the accumulation and metabolic capacity of photosynthetic carbohydrate products. This result confirmed the conclusion predicted by metabolomics. Tea polyphenol, caffeine and flavone all belong to secondary metabolites of tea tree, and this study found that the content of these indexes decreased significantly with the increase of Mg concentration, and the content of water extract and free amino acid also decreased significantly. It can be seen that Mg regulation was indeed detrimental to the improvement of most tea quality indexes. These results validated metabolomic predictions that Mg regulation was detrimental to the synthesis of secondary metabolites and amino acids related to tea quality.

## 4 Conclusion

**Figure 7:**
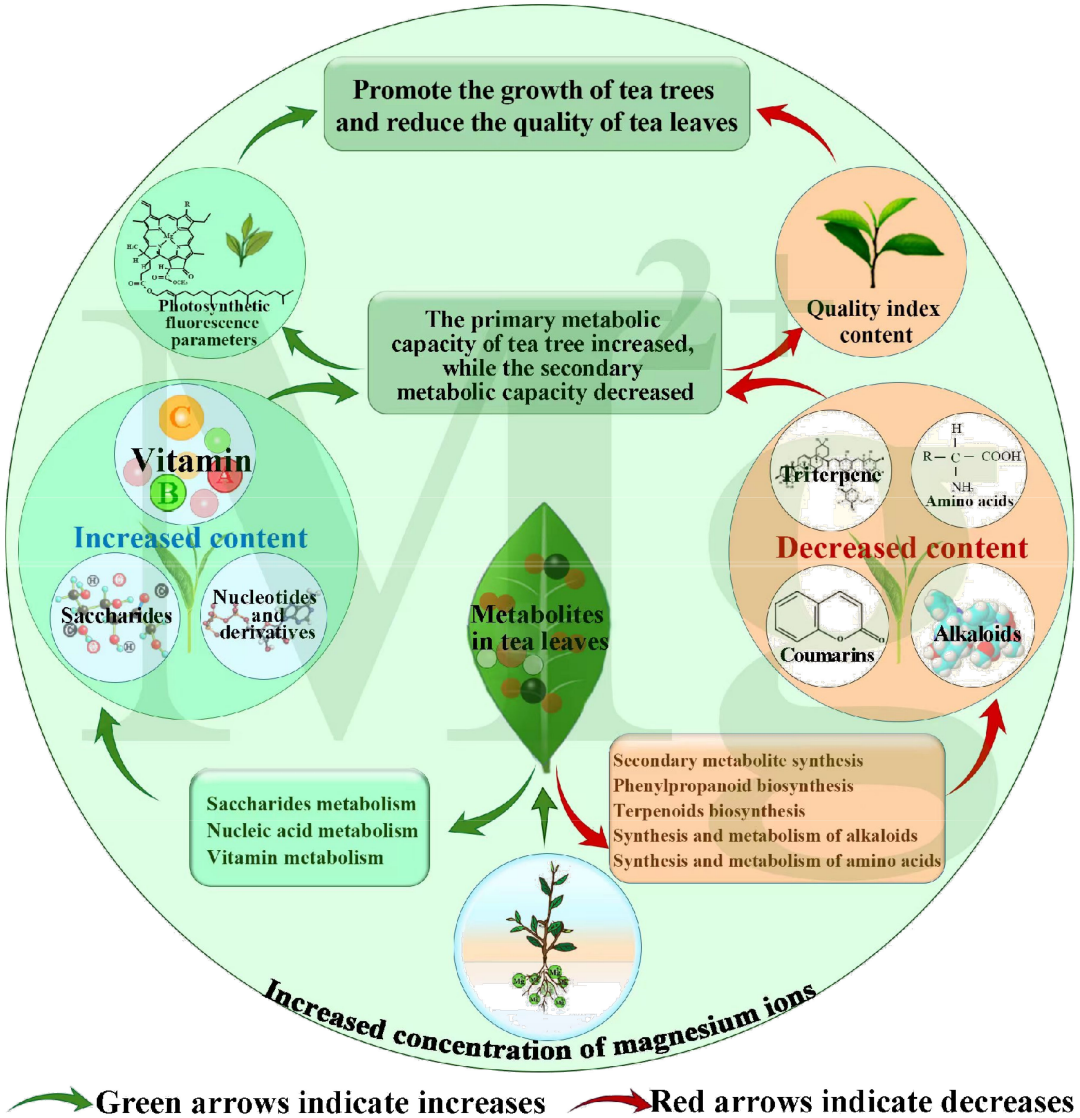
Mechanism of changes in metabolites of tea leaves under Mg regulation.

In this study, we analyzed the effect of magnesium regulation on metabolites of tea leaves and tea tree growth and quality. The results showed (**Figure 7**) that magnesium regulation was beneficial to enhance the photosynthetic capacity of tea trees, improve the accumulation and metabolism of carbohydrate substances in tea trees, and thus promote the growth of tea trees, but was not conducive to the synthesis of secondary metabolites and amino acids related to tea quality. The analysis results of photosynthetic fluorescence parameters and quality indexes of tea leaves showed that with the increase of magnesium treatment concentration, photosynthetic fluorescence parameters of tea leaves showed an increasing trend, and the contents of theanine and soluble sugar also showed an increasing trend, while the contents of tea polyphenols, caffeine, flavone, water extract and free amino acid showed a decreasing trend. This result validated the metabolomics predictions. This study provided some reference for regulating the growth and quality of tea trees with magnesium fertilizer in tea plantations.

## Data availability statement

The original contributions presented in the study are included in the article/**Supplementary Material**, further inquiries can be directed to the corresponding author/s.

## Author contributions

YZ and QZ: Conceptualization, Visualization, Methodology, Writing – original draft, Formal analysis, Writing – review & editing, Funding acquisition. YW and SL: Formal analysis, Writing – review & editing. MC and PC: Methodology, Investigation, Writing – original draft. MD and XJ: Methodology, Investigation. HW and JY: Conceptualization, Visualization, Methodology, Writing – original draft, Formal analysis, Writing – review & editing, Funding acquisition. All authors contributed to the article and approved the submitted version.
